# Adaptation of the methotrexate in rheumatoid arthritis knowledge questionnaire (MiRAK) for use with parents of children with juvenile idiopathic arthritis: a qualitative study

**DOI:** 10.1186/1546-0096-11-27

**Published:** 2013-07-03

**Authors:** Sadie Wickwar, Katrin Buerkle, Hayley McBain, Sabina Ciciriello, Richard H Osborne, Lucy R Wedderburn, Stanton P Newman

**Affiliations:** 1 Centre for Health Services Research, School of Health Sciences, City University, College Building, Room A224, Northampton Square, London, EC1V 0HB, UK; 2 Community Health Newham, East London Foundation Trust, London, UK; 3 UCL Institute of Child Health, 30 Guilford Street, London, WC1N 1EH, UK; 4 Department of Rheumatology, Royal Melbourne Hospital, Victoria, Australia; 5 Public Health Innovation, Population Strategic Research Centre, School of Health & Social Development, Deakin University, Victoria, Australia

**Keywords:** Juvenile idiopathic arthritis, Questionnaire adaptation, Methotrexate, Cognitive interviews, Parent measures

## Abstract

**Background:**

Although Methotrexate (MTX) is one of the most commonly prescribed disease-modifying drugs in JIA no questionnaire exists that assesses the knowledge of parents about this drug. A 60-item questionnaire was recently developed to measure rheumatoid arthritis (RA) patients’ knowledge about MTX; the Methotrexate in Rheumatoid Arthritis Knowledge Test (MiRAK; Ciciriello et al. (Arthritis Rheum 62:10–1009, 2010)). This study aimed to adapt the MiRAK for parents of children with JIA.

**Methods:**

Adaption of the MiRAK involved: 1) email consultations with clinicians working in the field of paediatric rheumatology (Panel 1) to ascertain the potential adaptations of the MiRAK from a clinical perspective, 2) synthesis of clinicians’ suggestions by a panel of experts, researchers and MiRAK developers (Panel 2) to reach consensus on which items needed to be modified and create a draft Methotrexate in Juvenile Idiopathic Arthritis Knowledge Test (MiJIAK), 3) a review of the draft by 5 parents of children with JIA (Panel 3) using the cognitive ‘think-aloud’ method, 4) a second consultation with Panel 2 to review parents’ suggestions and determine the final items.

**Results:**

A total of 9 items remained unchanged, e.g. “*Methotrexate is effective at relieving joint stiffness*”, 19 were deemed inappropriate in the paediatric setting and deleted, e.g. “*It is safe to become pregnant 3 weeks after methotrexate has been stopped*”, 32 underwent editorial changes largely to indicate that the questionnaire was about the children with JIA, e.g. “*If you forget to give a dose of Methotrexate*, *you can still take it the next day*” became “*If your child misses a dose of Methotrexate*, *they can still take it the next day*”, and 1 new item was added. A new 42-item questionnaire was produced and was found to be well understood by parents of children with JIA.

**Conclusions:**

The systematic modification of the MiRAK, a patient-centred MTX knowledge questionnaire, has generated a comprehensive new questionnaire for use in the JIA setting. The wide consultation process, including cognitive testing, has ensured the tool is both relevant and acceptable to clinicians and will therefore be a valuable addition in understanding the parents’ perspective of this treatment in JIA.

## Background

Juvenile idiopathic arthritis (JIA) is the most common rheumatic inflammatory disorder found in children, with an annual incidence of approximately 10 per 100,000 children [1]. The International League of Associations for Rheumatology (ILAR) criteria define JIA as occurring in children under the age of 16 years where arthritis in one or more joints persists for more than 6 weeks with unknown cause [2]. As there is currently no cure, medical intervention aims to manage the illness and its symptoms, principally pain, stiffness and joint swelling [3]. Methotrexate (MTX) is the most commonly used disease modifying drug (DMARD) in JIA and has been widely shown to generate considerable improvement in symptoms [3]. However, MTX is not effective for all children [4].

Parents play an important role in the management of their child’s illness and there are few chronic diseases as challenging for a child and their parents as severe JIA [5]. One major issue that concerns parents is the possible side effects of their child’s medication [6]. MTX is known to cause a number of unpleasant side effects, including nausea, vomiting, diarrhoea and mouth sores, and these can be distressing for both the child and parent [7] MTX is also used to treat cancer. This requires much higher doses and carries the risk of greater toxicity than low-dose MTX used for JIA. Parents may be alarmed by information about MTX that is not specific to JIA and lists side effects that do not occur with low-dose MTX treatment. Given the potential difficulties around the use of MTX for treating JIA, and the role parents play in supporting the management of the disease, it is important that parents receive accurate information from clinicians about MTX. Assessment of parents’ knowledge regarding medications in JIA reveals that this may be quite limited and consequently, educational interventions able to increase knowledge significantly are necessary [8].

It is important to have measures specifically designed for parents to capture the unique experiences of caring for a child with a chronic illness, and the knowledge they have about their child’s treatment. Questionnaires can provide a valid and reliable way to evaluate the effectiveness of education efforts [9] and can assist healthcare providers in determining how best to use resources such as staff time when developing education packages and assisting parents. However, there is no comprehensive, validated and reliable measure of MTX knowledge in parents of children with JIA. To date studies have used methods with limited reproducibility and validity [8] including unstructured observations and investigator-designed questions.

This study therefore aimed to adapt a robust measure of MTX knowledge for adult rheumatoid arthritis patients for use with parents of children with JIA. The Methotrexate in Rheumatoid Arthritis Knowledge (MiRAK) questionnaire was developed using grounded approaches involving both clinicians working in the field of rheumatology and patients with RA [10]. The questionnaire consists of 60 items that were based on the words and concepts used by people with the condition, clinical relevance, and the published literature. Respondents answer each statement as either ‘true’, ‘false’ or ‘don’t know’. Scores are calculated by adding together the number of correct responses. The MiRAK has been reported to have excellent psychometric properties including good model fit, supporting internal construct validity; good internal consistency (person separation index 0.84); test-retest reliability (ICC 0.89) and ability to detect change (Effect Size 2.38) [10].

## Methods

### Study design

This study adapted the MiRAK for use by parents of children with JIA using the Delphi technique, expert consensus, followed by cognitive think-aloud interviews with parents of children with JIA.

### The Delphi technique

We used the Delphi technique [11] to obtain feedback regarding which items to include in the revised tool. This process involved 3 panels of experts; a group of paediatric rheumatologists, a panel of researchers working in rheumatology and parents of the children with JIA. The Delphi method has been previously used in health services research [12,13], including the context of questionnaire construction and validation alongside the use of think aloud interviews [14].

### Think-aloud technique

The think-aloud technique involves asking a respondent to comment on a task while undertaking the task, thus providing rich verbal data on the experience of completing a tool. Think-aloud interviewing is an established method used in the adaptation and validation of questionnaires in health settings [15,16]. In the present study two interviewers instructed each parent to provide a running commentary on their thoughts about each questionnaire item using an interview schedule. Some probing questions such as “*what are your feelings about that item*?” were used to encourage parents to continue to think aloud, whilst contributions made by the interviewer during this process were kept to a minimum. Parents were also asked to make any suggestions for additional items they felt should be included.

### Participants

A convenience sample of 3 paediatric rheumatologists and one rheumatology nurse specialist was recruited to Panel 1. This type of sampling involves approaching only those available during the recruitment period. A second panel consisted of two authors of the MiRAK (a rheumatologist and a professor of public health), a professor in paediatric rheumatology, a professor in health psychology and 2 research assistants working in JIA. The third panel consisted of parents and was recruited via one centre (Great Ormond Street Hospital). Parents were invited to participate if they; [1] were either the mother or father of a child with JIA, [2] had experience of giving their child MTX, [2] were already taking part in the Childhood Arthritis Response to Medication Study (CHARMS), [3] had clinic appointments booked during the recruitment period for this study and [4] were able to understand and speak English. A total of 36 parents were identified as being eligible and were invited to take part in the study, of these 5 agreed to participate. Participating parents were also asked to complete a demographics questionnaire that recorded their age, employment status, level of education, their child’s JIA subtype, and the length of time since their child’s diagnosis. Members of Panel 3 were aged between 35 and 50 years. Two were full-time homemakers, two were full-time employed, and one did not disclose their occupation. All of the parents were married or lived with their partner, and all lived with their child who had JIA. The majority were educated to degree level with two parents educated to postgraduate level. The children of these parents either had extended oligoarthritis, polyarticular JIA or psoriatic JIA. All children were still taking MTX at the time of the study and had been living with the diagnosis of JIA for between 3 and 9 years.

### Data collection and analysis

Quantitative data were recorded at each of the following stages using descriptive statistics. Frequencies were used to examine the number of times suggestions were made about the retention, removal or adaptation of an item by both the paediatric rheumatologists and parents.

#### Stage 1

Members of Panel 1 were sent a pre-designed questionnaire adaptation form that consisted of instructions for clinicians and a list of all the original MiRAK items. There was space for clinicians to make suggestions for editing each item and the document automatically tracked the changes that they made. Members of Panel 1 were instructed to review each item of the MiRAK and recommend whether each item should “stay as it is”, “be deleted”, or “be reworded”. Respondents were also invited to suggest rewording. Responses were then coded by 2 researchers into one of the following groups “*modification of original MiRAK item*”, “*deletion of original MiRAK item*”, or “*original MiRAK item retained*”. Responses from all 4 members were then consolidated.

#### Stage 2

Results were presented to Panel 2 containing the responses from Panel 1 on each individual item. Considering these results Panel 2 then made decisions on the consistency of the responses across the 4 participants, the consistency of the wording between items, the relevance of the item to JIA and the accuracy of the content of each item. Initially, any items for which there was 100% agreement between the clinicians were retained. The remaining items were put forward for discussion by the members of Panel 2. The development of new items was based on the general rules and principles of item construction used in previous questionnaire development research [17]. Consensus was reached through discussion until a first version of the new questionnaire was agreed upon.

#### Stage 3

Following consent this draft questionnaire was then presented to parents of children with JIA within cognitive think-aloud interviews. These interviews lasted approximately one hour and each was recorded. Interviews with parents were transcribed and coded using the codes from stage 1 i.e. “*modification of item*”, “*deletion of item*”, “*item retained*”, or “*addition of new item*”. Where parents made comments about the acceptability of an item, these were coded either “*clear*”, “*acceptable*” or “*difficult to understand*”.

#### Stage 4

The input provided by the parents was then assessed by Panel 2 to create the final version of the questionnaire. If there was not 100% agreement between the parents in terms of retaining or adapting an item, the two research assistant members of the panel made suggestions about possible alternatives which were reviewed and agreed by the remaining members of the panel.

### Ethical approval

The study received approval from the GOSH Research Ethics Committee.

## Results and discussion

**Figure 1:**
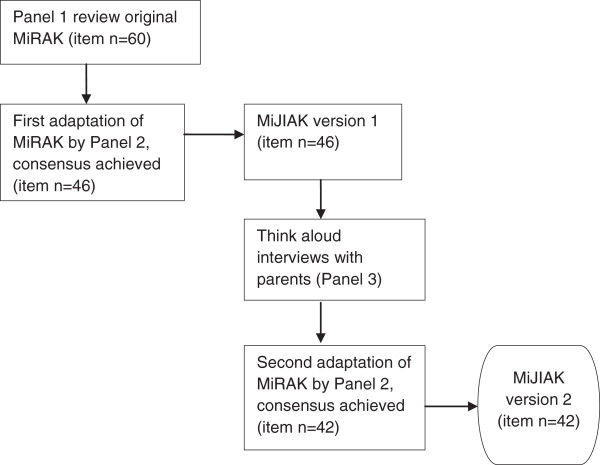
Results from each stage of the Delphi process.

Figure 1 summarizes the results obtained from each stage of the Delphi process.

### Stage 1: panel 1 item review

All 4 members of Panel 1 agreed that one of the MiRAK items needed to be removed from the questionnaire, as it was considered not relevant to JIA (“*Daily dose aspirin* (*one tablet or less a day*) *should not be taken while on methotrexate*” - False). Panel 1 also agreed on 11 items that needed to be reworded to make them relevant to JIA; the predominant change was minor (e.g. “*rheumatoid arthritis*” to “*juvenile idiopathic arthritis*”). There was consistent advice for 9 items to be retained as in the original questionnaire. The remaining items (n= 39) for which 100% agreement was not reached amongst the clinicians were reviewed by Panel 2.

### Stage 2: panel 2 consensus & MiJIAK first draft

Panel 2 removed an item originally retained by Panel 1 as it was not considered to have adequate evidence to provide a clear true/false response in JIA (“*Methotrexate can reverse joint damage caused by rheumatoid arthritis*” - False). Of the 39 items where Panel 1 did not reach consensus, 12 items were removed as they were found to be not relevant in JIA; a further 4 were retained as in the original questionnaire and the remaining items (n=23) were adapted by Panel 2 often with small amendments. (e.g. “*You should keep taking methotrexate even when your joints are not painful*” was changed to “*Your child should keep taking methotrexate even when their joints are not painful*”). As a result of this second process the original 60-item MiRAK was adapted to create the first draft of a 46-item Methotrexate in Juvenile Idiopathic Arthritis Knowledge (MiJIAK) questionnaire.

### Stage 3: parents’ think aloud interviews

Of the 46 items all parents agreed that 23 items were clear and acceptable. Parents queried the clarity of the wording of 4 items and the relevance of two items. For example, one parent said "*That I*'*d find quite worrying*…*people may think the liver*'*s going to go wrong*" in regards to the item “*Scarring of the liver is a common side effect of methotrexate*” - False. Parents also suggested 1 additional item to be included, “*There*'*s nothing in there about the best time to take MTX*, *and about food that should or shouldn*'*t be eaten*”. As a result a new item was added “*Methotrexate should be taken on the same day each week*” - True.

### Stage 4: panel 2 consensus & final version of MiJIAK

A second consultation took place with Panel 2 to review parents’ suggestions and to reach consensus on the inclusion and wording of the final items. Concerns expressed by parents on the item regarding scarring of the liver resulted in it being removed. The medical accuracy of two items for their use in the case of JIA was further queried by the panel and this resulted in them being removed because of lack of clear evidence in JIA (e.g. “*Methotrexate does not slow the joint damage caused by rheumatoid arthritis*” - False) Four additional items were removed from the final version as parents felt there was either a lack of clarity or the question was not relevant to children. (e.g. “*If you are unable to eat or drink you should still try to take your methotrexate*” – False)

In total, 9 items remained unchanged, 19 were deleted, the wording of 32 was changed, and 1 new item was added. The final version of the MiJIAK questionnaire has 42 items and includes a variety of ‘true’ and ‘false’ response scores (29 and 13 respectively). The 42-item questionnaire takes 15 minutes to complete. An additional file shows the questionnaire [see Additional file 1].

### Readability

To determine the reading level of the questionnaire, the Flesch-Kincaid Reading Ease and Flesch-Kincaid Grade Level tests [18] were performed on the final version of the MiJIAK, including the instructions and each of the 42 items of the questionnaire. A Flesch-Kincaid Reading Ease score of 49 (higher scores out of 100 indicate easier readability) and a Flesch-Kincaid Grade Level of 10 (i.e. 15 to 16 years old) were found.

## Discussion

The adaption of the MiRAK for use with parents of children with JIA was achieved through the use of consultations with clinicians to identify necessary changes from a medical perspective and researchers to ensure clarity and consistency. These consultations were followed by cognitive think-aloud interviews with parents of children with JIA to obtain opinions from the target population about the clarity and acceptability of the new questionnaire. This resulted in the new 42-item Methotrexate in Juvenile Idiopathic Arthritis Knowledge Test (MiJIAK), which parents found clear to understand and was acceptable to clinicians in the field.

This is the first questionnaire designed to measure MTX knowledge specifically in parents of children with JIA. Such a questionnaire can be used in the context of JIA to highlight the educational needs of an individual or population to ensure knowledge from the parent’s perspective. Ensuring that parents have good knowledge lays the foundation for appropriate administration of medication and potentially reduces anxiety around MTX and its administration. The questionnaire can also be used to evaluate the effectiveness of educational interventions undertaken with parents responsible for administering their child’s MTX.

The think-aloud interviews permit the gathering of critical data about how to adapt the MiRAK using the immediate and honest thoughts of parents. Involving the target group in this way generated insight into the appropriateness of the MiJIAK for use with parents and ensured that the questionnaire had good content validity.

The Delphi method used offered a rigorous process by which to distribute and process data and to reach consensus about which items to include in the revised questionnaire and how best to word them. This method also allowed discussions to take place between members of the panel that could not be present physically, such as those based in Australia.

There are however some limitations in the study’s design. Firstly, the think-aloud interviews generated varying amounts of data from parents. The technique as described by Willis 1999; [19] involves a process of participants engaging with the research by providing a running commentary of their thoughts about a particular topic, which assumes participants are highly cognitively able and can be successfully engaged. The abilities of some of the parents may not have met these requirements, thus generating data that differed in both quantity and quality.

Secondly, any conclusions that were made about adapting specific questionnaire items were based on the suggestions of a small sample of mostly well educated middle aged adults, which may not have been representative of the population. A larger, more diverse sample may have been more appropriate. The questionnaire was found to be suitable for the reading level of 15 to 16 year olds, which may be limiting for some respondents.

Thirdly, this adaptation study does not include the validation and field testing of the revised questionnaire. Further testing in a variety of settings will provide additional data on the validity and reliability of the questionnaire and thus generate data regarding the utility of the tool [20]. Given the rigorous development of the tool, including a strong reliance on the most recent published literature and input from experienced rheumatologists from both the UK and Australia, the tool has very good face and content validity and is likely to reveal important information about the accuracy of the knowledge of parents of children with JIA.

## Conclusions

The 42-item MiJIAK is the first MTX knowledge questionnaire designed specifically for parents of children with JIA and can be used to generate insight into the information needs, the quality of information provided to parents, and the outcome of education interventions in this field. Prior to this however, further research to explore the validity and reliability of the revised measure across settings will facilitate the MiJIAK’s application into the research and clinical setting.

## Abbreviations

JIA: Juvenile idiopathic arthritis; MTX: Methotrexate; RA: Rheumatoid arthritis; MiRAK: Methotrexate in Rheumatoid Arthritis Knowledge Test; MiJIAK: Methotrexate in Juvenile Idiopathic Arthritis Knowledge Test; GOSH: Great Ormond Street Hospital

## Competing interests

The authors declare that they have no competing interests.

## Authors’ contributions

SW contributed to data collection, coordination of the study, data analysis and interpretation, and compiled the first manuscript draft. KB coordinated the study and participated in the design of the study, data collection, data analysis, interpretation and drafting the final manuscript. HM helped to draft the final manuscript. RO, SC and SN were involved in data analysis, data interpretation and drafting the final manuscript. LW and SN conceived the study and LW coordinated clinicians who participated in the study. All authors read and commented on the manuscript.

## Supplementary Material

Additional file 1 Methotrexate in Juvenile Idiopathic Arthritis Knowledge test (MiJIAK). Click here for file
