# Supplementary material for: Adaptation of the methotrexate in rheumatoid arthritis knowledge questionnaire (MiRAK) for use with parents of children with juvenile idiopathic arthritis: a qualitative study
Source: Pediatr Rheumatol Online J. 2013 Jul 3;11:27. doi: 10.1186/1546-0096-11-27 (PMC3704946; doi:10.1186/1546-0096-11-27)
Supplement: Additional file 1 — Methotrexate in Juvenile Idiopathic Arthritis Knowledge test (MiJIAK). [file 1546-0096-11-27-S1.pdf]

Additional File 1: Methotrexate in Juvenile Idiopathic Arthritis Knowledge test (MiJIAK)

Instructions:

The following statements are about Methotrexate and Juvenile Idiopathic Arthritis (JIA). Please mark whether you think these statements are true or false. These questions are testing the quality of the information you have been given. We therefore ask that if you do not know the answer to a question you mark "don't know" rather than trying to guess the answer or looking it up. Please answer all of the questions.

Q1	At present there is no cure for juvenile idiopathic arthritis	TRUE		FALSE		DON'T KNOW	
Q2	Methotrexate is effective at relieving joint stiffness	TRUE	☐	FALSE	☐	DON'T KNOW	☐
Q3	Taking regular folic acid (also known as folate, folicare or lexpec) reduces the chance of getting side effects from methotrexate	TRUE		FALSE		DON'T KNOW	
Q4	Methotrexate can cause mouth ulcers	TRUE	☐	FALSE	☐	DON'T KNOW	☐
Q5	The low dose of methotrexate used to treat juvenile idiopathic arthritis has the same risks and side effects as the higher doses used to treat other conditions	TRUE		FALSE		DON'T KNOW	
Q6	Drinking alcohol increases the chance of getting liver damage from methotrexate	TRUE	☐	FALSE	☐	DON'T KNOW	☐
Q7	If your child misses a dose of methotrexate, they can still take it the next day	TRUE		FALSE		DON'T KNOW	
Q8	Methotrexate tablets are white	TRUE	☐	FALSE	☐	DON'T KNOW	☐
Q9	Methotrexate is also used to treat cancer	TRUE		FALSE		DON'T KNOW	
Q10	The antibiotic Trimethoprim (also known as Trimopan) can be safely taken while on methotrexate	TRUE	☐	FALSE	☐	DON'T KNOW	☐

Q11	Most children and young people can safely take methotrexate for years	TRUE		FALSE		DON'T KNOW	
Q12	Juvenile idiopathic arthritis causes joint inflammation	TRUE	☐	FALSE	☐	DON'T KNOW	☐
Q13	Regular folic acid (also known as folate, folicare or lexpec) should be taken by most children who are taking methotrexate	TRUE		FALSE		DON'T KNOW	
Q14	The benefits of methotrexate should be noticed within a few days of starting it	TRUE	☐	FALSE	☐	DON'T KNOW	☐
Q15	Nausea is a common side effect of methotrexate	TRUE		FALSE		DON'T KNOW	
Q16	Treatment of juvenile idiopathic arthritis with medications can prevent joint damage	TRUE	☐	FALSE	☐	DON'T KNOW	☐
Q17	Methotrexate can cause young women to have irregular periods	TRUE		FALSE		DON'T KNOW	
Q18	Taking regular folic acid (also known as folate, folicare or lexpec) reduces joint pain and swelling	TRUE	☐	FALSE	☐	DON'T KNOW	☐
Q19	Methotrexate is a commonly used treatment for juvenile idiopathic arthritis	TRUE		FALSE		DON'T KNOW	
Q20	Treatment for juvenile idiopathic arthritis is more effective if it is started early	TRUE	☐	FALSE	☐	DON'T KNOW	☐
Q21	Your child needs to continue having regular blood tests as long as they take methotrexate	TRUE		FALSE		DON'T KNOW	
Q22	Anti-inflammatory medications (for example Nurofen or Ibuprofen) should not be used while taking methotrexate	TRUE	☐	FALSE	☐	DON'T KNOW	☐
Q23	If your child misses a dose of methotrexate, you should double the dose next time to make up for it	TRUE		FALSE		DON'T KNOW	

Q24	Methotrexate is often combined with other medications used to treat juvenile idiopathic arthritis	TRUE	☐	FALSE	☐	DON'T KNOW	☐
Q25	Your child should not have the flu vaccine while on methotrexate	TRUE	☐	FALSE	☐	DON'T KNOW	☐
Q26	Both young men and women taking methotrexate should use reliable birth control (contraception)	TRUE	☐	FALSE	☐	DON'T KNOW	☐
Q27	Anti-inflammatory medications (for example Nurofen or Ibuprofen) can slow joint damage caused by juvenile idiopathic arthritis	TRUE	☐	FALSE	☐	DON'T KNOW	☐
Q28	Methotrexate should be stopped if your child's juvenile idiopathic arthritis flares up (becomes worse)	TRUE	☐	FALSE	☐	DON'T KNOW	☐
Q29	Methotrexate can cause thinning of the hair	TRUE	☐	FALSE	☐	DON'T KNOW	☐
Q30	All medications used to treat juvenile idiopathic arthritis can cause side effects	TRUE	☐	FALSE	☐	DON'T KNOW	☐
Q31	Any young person who drinks alcohol should restrict their alcohol intake while taking methotrexate	TRUE	☐	FALSE	☐	DON'T KNOW	☐
Q32	Your child should take methotexate daily (tablets, syrup or injections)	TRUE	☐	FALSE	☐	DON'T KNOW	☐
Q33	Your child should keep taking methotrexate even when their joints are not painful	TRUE	☐	FALSE	☐	DON'T KNOW	☐
Q34	Methotrexate is effective at relieving joint swelling	TRUE	☐	FALSE	☐	DON'T KNOW	☐
Q35	Treatment for newly diagnosed juvenile idiopathic arthritis often includes methotrexate	TRUE	☐	FALSE	☐	DON'T KNOW	☐
Q36	The chance of side effects increases when methotrexate is taken more often than prescribed	TRUE	☐	FALSE	☐	DON'T KNOW	☐

Q37	Your child should not have any live vaccinations while on methotrexate	TRUE		FALSE		DON'T KNOW	
Q38	Blood tests are done to pick up side effects caused by methotrexate	TRUE	☐	FALSE	☐	DON'T KNOW	☐
Q39	Methotrexate treatment will be stopped once your child's arthritis is under control	TRUE		FALSE		DON'T KNOW	
Q40	Methotrexate should be stopped if your child develops a cold	TRUE	☐	FALSE	☐	DON'T KNOW	☐
Q41	Methorexate tablets come in two different strengths	TRUE		FALSE		DON'T KNOW	
Q42	Methotrexate should be taken on the same day each week	TRUE	☐	FALSE	☐	DON'T KNOW	☐
